# Prediction of Gap Asymmetry in Differential Micro Accelerometers

**DOI:** 10.3390/s120606857

**Published:** 2012-05-25

**Authors:** Wu Zhou, Baili Li, Bei Peng, Wei Su, Xiaoping He

**Affiliations:** 1 School of Mechatronics Engineering, University of Electronic Technology and Science of China, Chengdu 611731, China; E-Mail: beipeng@uestc.edu.cn; 2 School of Mechanical Engineering, University of Southwest Jiaotong University, Chengdu 610031, China; E-Mail: blli62@263.net; 3 Institute of Electronic Engineering, China Academy of Engineering Physics, Mianyang 621900, China; E-Mails: weisu@caep.ac.cn (W.S.); hexpiee@263.net (X.H.)

**Keywords:** MEMS, accelerometer, asymmetry, analytical method

## Abstract

Gap asymmetry in differential capacitors is the primary source of the zero bias output of force-balanced micro accelerometers. It is also used to evaluate the applicability of differential structures in MEMS manufacturing. Therefore, determining the asymmetry level has considerable significance for the design of MEMS devices. This paper proposes an experimental-theoretical method for predicting gap asymmetry in differential sensing capacitors of micro accelerometers. The method involves three processes: first, bi-directional measurement, which can sharply reduce the influence of the feedback circuit on bias output, is proposed. Experiments are then carried out on a centrifuge to obtain the input and output data of an accelerometer. Second, the analytical input-output relationship of the accelerometer with gap asymmetry and circuit error is theoretically derived. Finally, the prediction methodology combines the measurement results and analytical derivation to identify the asymmetric error of 30 accelerometers fabricated by DRIE. Results indicate that the level of asymmetry induced by fabrication uncertainty is about ±5 × 10^−2^, and that the absolute error is about ±0.2 μm under a 4 μm gap.

## 1. Introduction

The development of micro-electro-mechanical-systems (MEMS) usually involves the fabrication of small structures with relative errors larger than that observed in traditional fabrication technology. Therefore, evaluating the level of structural error and its influence in micro-fabrication has significance in the design of new devices and improvement of processes. Given the immaturity and diversity of micro fabrication techniques, researchers and institutions adopt evaluation methods with focus and specificities particular to such approaches. For example, Cigada used the electrical method to measure the dynamic behaviors of a MEMS gyroscope with fabrication error [1]; Wittwer predicted the error effect on compliant mechanisms from the perspective of dimensions and materials [2]; and Pugno suggested a novel method for predicting the strength of microstructures with complex geometries that arise from micro processes [3]. Aside from direct measurements or prediction methods, design methodologies have been proposed to reduce the dependence of device performance on microfabrication errors; these methodologies include optimization [4–7] and robust design technologies [8–10]. Error prediction in the current work was indirectly carried out to evaluate gap asymmetry in differential capacitors of micro accelerometers fabricated by deep reactive-ion etching (DRIE). DRIE is a highly anisotropic etching process used to create microstructures with high aspect ratios; it is extensively used to fabricate micro accelerometers [11], micro gyroscopes [12,13], micro switches [14–16], micro actuators [17], micro gears [18] and so on. Gap asymmetry pertains to the disproportion between the capacitive gaps of two capacitors that form a differential sensor. Its occurrence causes the calibration line to deviate from its origin and forms zero bias output; that is, the output voltage of a force-balanced accelerometer is no longer zero under zero acceleration input [19,20]. Consequently, the measurement ranges in the positive and negative directions differ, and feedback circuit parameters cannot be established in a similar manner as those designed under ideal conditions. Thus, identifying the level of asymmetry in structures is urgently needed before differential sensors with new performance requirements can be designed.

The rest of the paper is structured as follows: Section 2 presents the centrifuge tests conducted in this study, and the observations of zero bias in accelerometers fabricated by DRIE. Section 3 discusses the prediction process, including the analytical derivation and reverse calibration method. Finally, Section 4 provides the results of the proposed experimental–theoretical method.

## 2. Asymmetric Phenomenon during Testing

**Figure 1.**
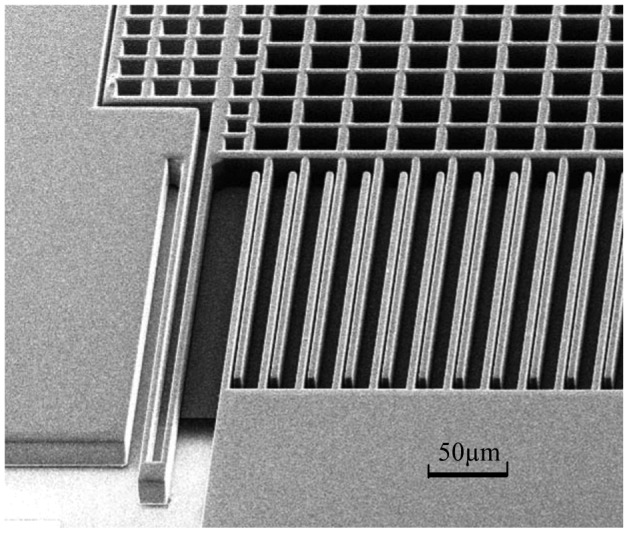
SEM of the sensor's structure.

Micro accelerometers are fabricated using DRIE bulk silicon technology and silicon bonding technology to provide high sensitivity and large signal output. These components have been proven successful in MEMS devices. The structure of the micro accelerometer used in this study is shown in Figure 1, which presents only a quarter of the entire structure. It consists of a folded beam, comb capacitors, and a sensing mass block. Accelerometer tests and calibration are performed on a centrifuge, which can simulate the acceleration field using a designed rotation speed. In the testing process, the sensitive direction of the accelerometer is along the radius of the circumrotating platform, thus the acceleration input is due to the centripetal acceleration resulting from uniform circular motion of the centrifuge platform. The range of acceleration input in this paper is between−55 G (where G is the acceleration of gravity) and +55 G, and the increment of test points is 5 G. We call the proposed experiment the bi-directional measurement method, which involves two independent processes intended to separate the influence of the feedback circuit from the structure gap error on bias output.

**Figure 2.**
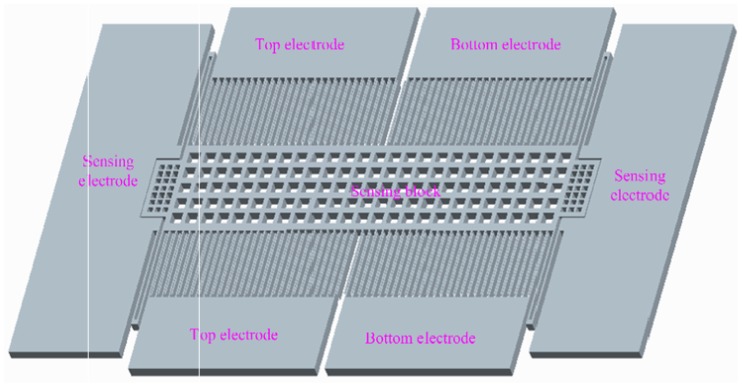
Differential sensor.

“Bi-” refers to two opposite ways of loading bias input voltage on the electrodes of the capacitors. For the positive direction, the top electrode shown in Figure 2 is connected to the positive voltage +*V* and the bottom electrode is connected to a voltage −*V*. For the negative direction, the power polarity is reversed (*i.e.*, the top electrode is connected to −*V* and the bottom electrode is connected to +*V*).

**Figure 3.**
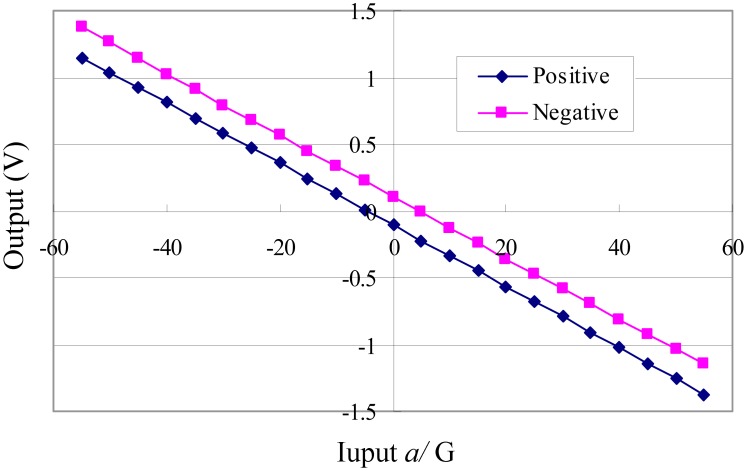
Data of experiments on the selected sensor.

The data are depicted in Figure 3. The experimental data indicate that the output voltage of the accelerometer under zero input is nonzero in both directions. This output is generally called zero bias, which is originally influenced by the asymmetry in the accelerometers. The asymmetry is caused by fabrication error and the zero error arising from the differential feedback circuit.

## 3. Prediction Method

**Figure 4.**
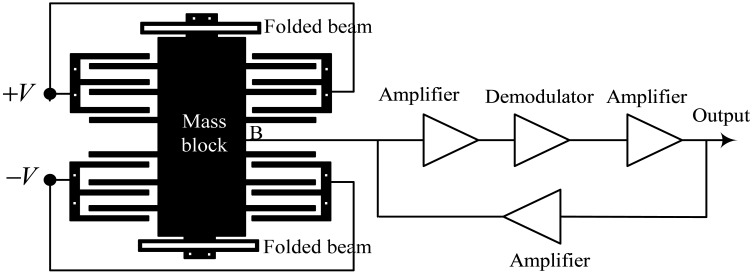
Diagram of an accelerometer.

Identifying the effect of asymmetric error on the accelerometer requires deriving and analyzing the operation principle of the sensor. The diagram of a typical differential accelerometer structure is shown in Figure 4. The structure comprises three core components: a movable mass block that converts acceleration into an inertial force, a differential capacitor structure for driving and sensing, and an electric circuit for feedback and signal output. The movable mass is suspended by folded beams at each end and deflects in the plane of the substrate under applied acceleration. A voltage signal is induced on the movable beams as a result of the change in differential capacitance caused by the movement of mass. Instantaneously, this signal is demodulated and amplified to yield a feedback voltage, which is applied to the movable beams via a feedback circuit. Consequently, an electrostatic force is generated, which drags the movable mass back to the zero position, and the accelerometer output is constituted by the amplified version of the feedback voltage. Under ideal conditions, the two groups of comb fingers that form the differential capacitors have similar overlap plate areas and gap distances. The output is zero under an ideal sensing circuit and zero input. In the actual engineering test, however, a zero bias output is generated because of fabrication error and circuit bias, discussed in the succeeding sections.

### 3.1. Analytical Relationship

**Figure 5.**
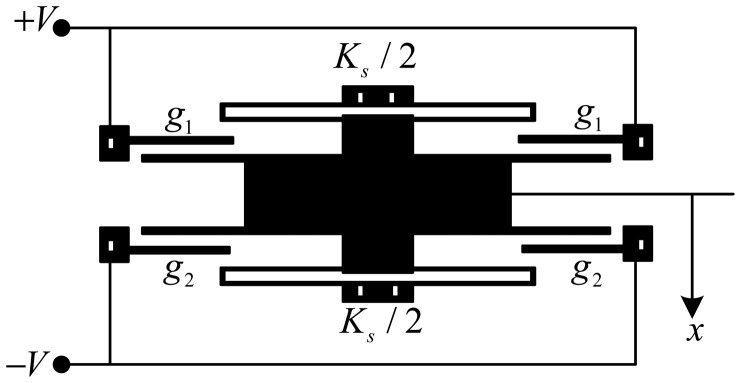
Simplified structure with average gaps.

The gap asymmetry caused by the micro fabrication is considered first. Its equivalent value is represented by the average value, which equals the ratio of the sum of the capacitor gaps and gap number (Figure 5). *g*_1_ and *g*_2_ are the average values of the top and bottom electrode gaps, respectively. *g*_1_ ≠ *g*_2_ reflects the asymmetry from the fabrication. When the bias voltages are applied to the fixed plates of the capacitors, the mass block moves to a position where the mechanical force equals the electrostatic force under closed-loop control.

Assuming that the sensing circuit is of an ideal state, the instantaneous feedback voltage on the block subjected to bias voltage can be expressed as:

(1) $$V_{ft}=M\frac{C_{1}-C_{2}}{C_{1}+C_{2}}V_{a}=\frac{M(g_{2}-g_{1})}{g_{2}+g_{1}}V_{a}$$

where *V_a_* is the amplitude of the applied bias AC voltage on the electrodes, *M* denotes the gain value of the closed-loop circuit resulting from the buffer, demodulator, and operational amplifier in the stable state [21], and *C*_1_ and *C*_2_ are the total capacitance values of the top and bottom capacitors, respectively.

When a voltage is applied, the mass block moves because of the asymmetry induced by the electrostatic force produced by the top and bottom capacitors. The movement deforms the supporting beams. The resultant force of the mass block can therefore be expressed as:

(2) $$F_{r}=-K_{s}x-F_{e1}+F_{e2}$$

where *K_s_* is the total stiffness of the supporting folded beams, *x* represents the coordinate, and *F_e_*_1_ and *F_e_*_2_ denote the electrostatic forces formed by two groups of capacitors, which can be expressed as follows:

(3) $$F_{e1}=\frac{1}{2}\frac{ɛ{ɛ}_{0}A{\left(V_{d}-V_{f}+V_{a}\sin(\omega t)\right)}^{2}}{{\left(g_{1}+x\right)}^{2}}$$

(4) $$F_{e2}=\frac{1}{2}\frac{ɛ{ɛ}_{0}A{\left(V_{d}+V_{f}+V_{a}\sin(\omega t)\right)}^{2}}{{\left(g_{2}-x\right)}^{2}}$$

where *ε*_0_ denotes the vacuum permittivity, *ε* is the relative dielectric constant, *A* is the effective overlap area of the plates, *V_d_* represents the DC bias voltage, and *V_f_* is the feedback voltage from the closed-loop control circuit, which can be expressed thus:

(5) $$V_{f}=\frac{M(g_{2}-g_{1}-2x)}{g_{2}+g_{1}}V_{a}$$

The balance position of the mass block is where the resultant force is zero, *F_r_* = 0, and the values of the average gaps under balance can be obtained by solving Equations (2)–(5).

To simplify, we set one average value *g*_1_ = *g*_0_, and the other average value *g*_2_ = *g*_0_ + *e*, where *e* is the asymmetric error from micro fabrication. Thus, the expressions of electrostatic force become:

(6) $$F_{e1}=\frac{1}{2}\frac{ɛ{ɛ}_{0}A{\left(V_{d}-V_{f}+V_{a}\sin(\omega t)\right)}^{2}}{{\left(g_{0}+x\right)}^{2}}$$

(7) $$F_{e2}=\frac{1}{2}\frac{ɛ{ɛ}_{0}A{\left(V_{d}+V_{f}+V_{a}\sin(\omega t)\right)}^{2}}{{\left(g_{0}+e-x\right)}^{2}}$$

where *e* is the asymmetric error. Feedback voltage *V_f_* becomes:

(8) $$V_{f}=\frac{M(e-2x)}{2g_{0}+e}V_{a}$$

The following definitions are introduced for simplification:

(9) $$\beta =\frac{V_{f}}{V_{a}}=\frac{M(e-2x)}{2g_{0}+e}$$

(10) $$X=\frac{x}{g_{0}}=\frac{E}{2}=\frac{M_{0}(2+E)}{2MV_{a}}V_{\text{out}}$$

where *M*_0_ is a constant, *V*_out_ is the output voltage, and *E* = *e*/*g*_0_.

When the frequency of the driving voltage is considerably larger than the natural frequency of the mass block, the electrostatic forces can be calculated using the effective value of the voltage. According to the law of action and reaction, when the external acceleration is applied to the accelerometer, inertial force *F_a_* must equal the resultant force of the electrostatic force and mechanical force *F_r_*, with the signs reversed. Thus, we obtain:

(11) $$F_{a}=-F_{r}=\frac{K_{s}g_{0}}{2}\left(2X+\frac{R\left({\left(1-\beta G_{a}\right)}^{2}+0.5G_{a}^{2}\right)}{{\left(1+X\right)}^{2}}-\frac{R\left({\left(1+\beta G_{a}\right)}^{2}+0.5G_{a}^{2}\right)}{{\left(1+E-X\right)}^{2}}\right)$$

where *G_a_* is the ratio of the AC and DC biases, *G_a_* = *V_a_*/*V_d_*, *R* = *K_e_*/*K_s_*, and *K_e_* = *εε*_0_*AV_d_*^2^*g*_0_^−3^.

Therefore, the input acceleration can be expressed as a function of output voltage:

(12) $$a=A_{g}+B_{g}V_{\text{out}}+C_{g}V_{\text{out}}^{2}+D_{g}V_{\text{out}}^{3}+\cdots$$

where the coefficients under *M* ≫ 1 can expressed as:

(13) $$A_{g}=\frac{K_{s}g_{0}E}{2m_{s}}$$

(14) $$B_{g}=\frac{-K_{s}g_{0}(16RMG_{a}-8RG_{a}^{2}-16R+8+12E)}{2m_{s}MM_{0}V_{a}{\left(2+E\right)}^{2}}$$

(15) $$C_{g}=0$$

(16) $$D_{g}\approx \frac{8K_{s}g_{0}R(G_{a}^{2}M^{2}-3G_{a}M+G_{a}^{2}+2)}{m_{s}{\left(2+E\right)}^{2}M^{3}M_{0}^{3}V_{a}^{3}}$$

where *m_s_* is the mass of the mass block. *C_g_* = 0 indicates that the monotonicity of the output–input relationship remains in the sensor, even though asymmetry exists.

We conclude that the presence of *E* yields a nonzero constant term *A_g_* in Equation (12). Hence, the bias output voltage is nonzero when the input acceleration is zero. Another cause of bias output is the presence of deviation *V_b_* in the differential sensing circuit. Assuming that the mechanical structure of the sensor is ideally symmetric, the feedback voltage applied to the mass block is:

(17) $$V_{f}=V_{b}-MXV_{a}$$

where *V_b_* is the deviation of the sensing circuit under zero input.

According to the preceding derivation, the input acceleration can be expressed as:

(18) $$a=\frac{K_{s}g_{0}}{m_{s}}\left(X-\frac{2R\left(\left(G_{b}+X-MG_{a}X\right)\left(1+G_{b}X-MG_{a}X^{2}\right)+G_{a}^{2}X\right)}{{\left(1-X^{2}\right)}^{2}}\right)$$

where *G_b_* = *V_b_*/*V_d_*.

The output voltage is:

(19) $$V_{\text{out}}=M_{0}V_{f}=M_{0}(V_{b}-MXV_{a})$$

Therefore, the input can be expressed in terms of the output as follows:

(20) $$a=A_{c}+B_{c}V_{\text{out}}+C_{c}V_{\text{out}}^{2}+\cdots$$

where the coefficients under *M* ≫ 1 can be expressed as:

(21) $$A_{c}\approx \frac{K_{s}g_{0}V_{b}}{m_{s}MV_{a}}\left(1-2R\left(1+\frac{V_{a}V_{b}}{V_{d}^{2}}\right)\right)$$

(22) $$B_{c}\approx \frac{2K_{s}g_{0}R}{m_{s}M_{0}V_{d}}$$

(23) $$C_{c}\approx \frac{2K_{s}g_{0}}{m_{s}M^{2}M_{0}^{2}V_{a}^{2}}(4RG_{b}+RG_{b}(2-MG_{a}))$$

We conclude that the existence of a circuit deviation also leads to bias output voltage because of the nonzero constant term *A_c_* induced by nonzero *V_b_* in Equation (21).

The influence of the structure and circuit error has been analyzed under deep feedback control. Gain value *M* is more than four orders of 10, resulting in two consequences: the mass block moves along a linear region under low acceleration input, and the influence of the gap asymmetry of the sensor structure is significantly larger than that of the circuit bias. Thus, the relationship can be expressed as:

(24) $$a\approx \frac{K_{s}g_{0}E}{2m_{s}}-\frac{8K_{s}g_{0}R}{m_{s}M_{0}V_{d}{\left(2+E\right)}^{2}}V_{\text{out}}$$

The conclusion derived from Equation (24) is characterized by two aspects: First, the first term on the right indicates the effect of the asymmetric error on the bias acceleration at zero point. Its value is equal to the distance from the cross point of the relationship curve and axis *V*_out_ = 0 to the original point. It is also independent of the parameters of the closed-loop control circuit. Second, the second term on the right indicates how the asymmetric error influences the linear constant of the calibration function.

### 3.2. Reverse Calibration

Reverse calibration pertains to input acceleration calibrated as a function of output voltage. According to the analytical procedure, formulating the input function of the output is more direct and clear than formulating the output function of the input. Thus, reverse calibration is introduced to study the gap asymmetric error resulting from micro fabrication. The results of the reverse calibration based on the least-squares method are:

(25) $$a_{p}\approx -4.63329-43.53494V_{\text{outp}}$$

(26) $$a_{n}\approx 4.64157-43.53899V_{\text{outn}}$$

where *V_outp_* and *V_outn_* are the output voltages of positive direction and negative direction, respectively; *a_p_* and *a_n_* are the input accelerations of positive direction and negative direction, respectively.

**Figure 6.**
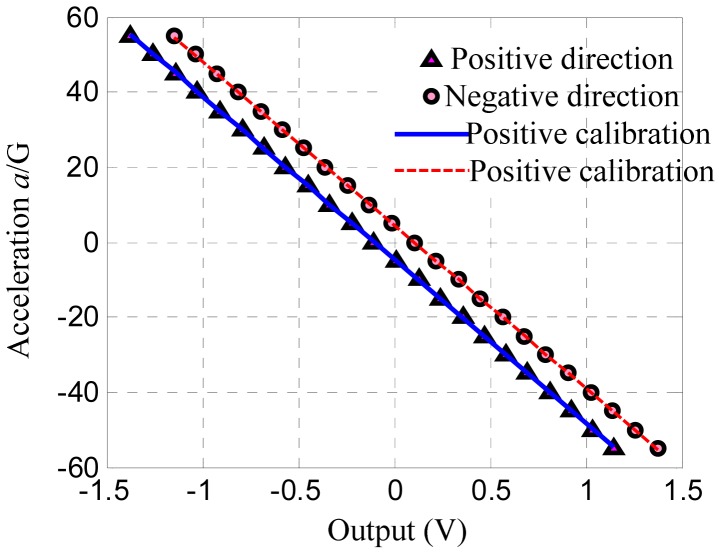
The test results in two directions.

**Figure 7.**
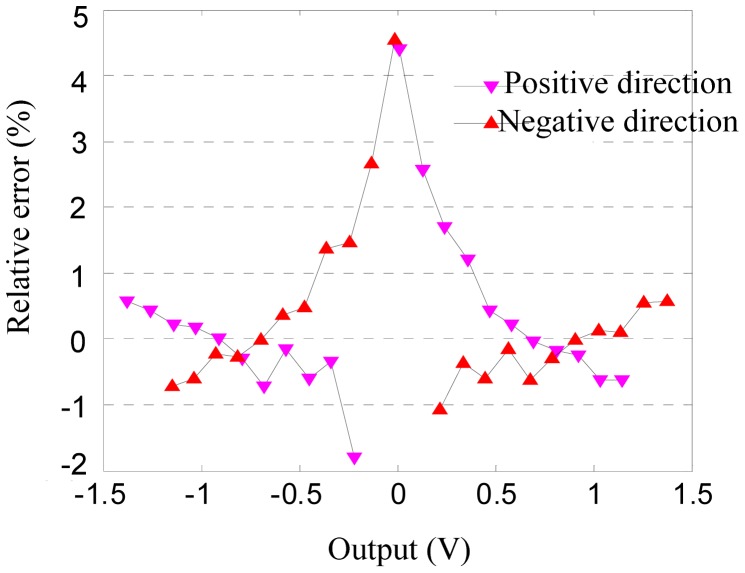
Relative error of calibration.

Figure 6 shows the reverse calibration curve lines of the experimental data. The plot directly indicates that the output voltage is nonzero when the acceleration is zero because of the asymmetric error. Figure 7 illustrates the relative errors of calibration, which indicate that the errors are less than 4.5%.

### 3.2. Prediction of Asymmetric Error

The theoretical–experimental prediction is established on the analytical and calibration functions. The ratio of constant term to linear term coefficient is selected in predicting the error, and can be expressed as:

(27) $$r_{a}=\frac{A_{g}}{B_{g}}=\frac{-K_{s}g_{0}^{3}M_{0}E{\left(2+E\right)}^{2}}{16ɛAV_{d}}=\frac{-V_{d}M_{0}E{\left(2+E\right)}^{2}}{16R}$$

**Figure 8.**
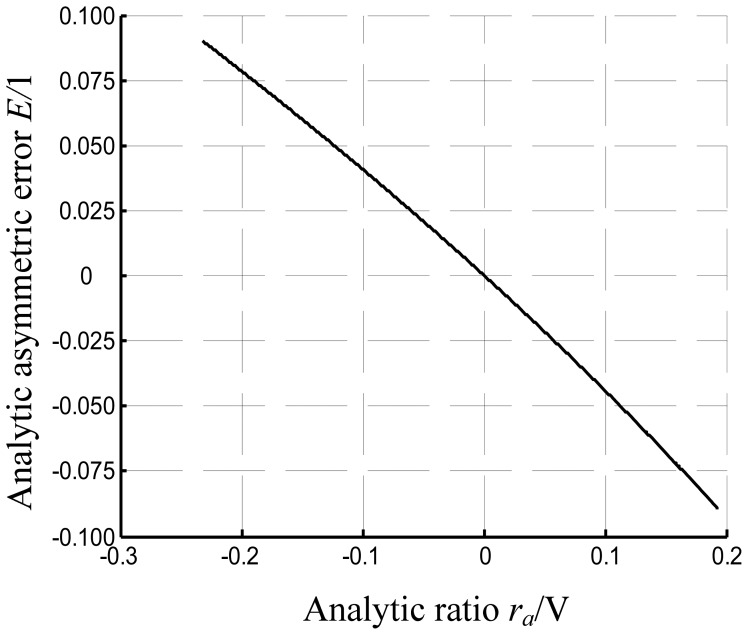
Asymmetric error *vs.* ratio in analytical relationship.

The conclusion derived from Equation (27) is that the ratio is now independent of the mass of the sensing block, AC bias, and gain value of the closed loop. The relationship between the error and ratio is depicted in Figure 8, and the parameters of the system are given in Table 1.

The reverse calibration result of a selected sensor leads to the coefficient ratio:

(28) $$r_{a}=\frac{-4.63329}{-43.53494}=0.10643$$

**Figure 9.**
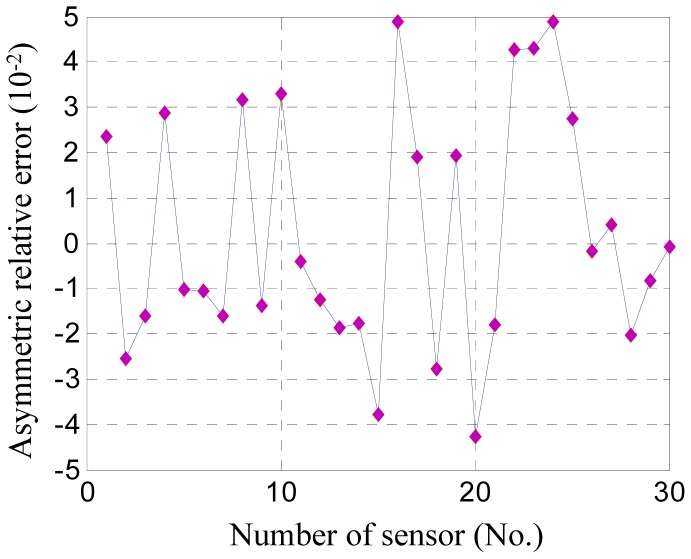
The prediction results of thirty sensors.

**Figure 10.**
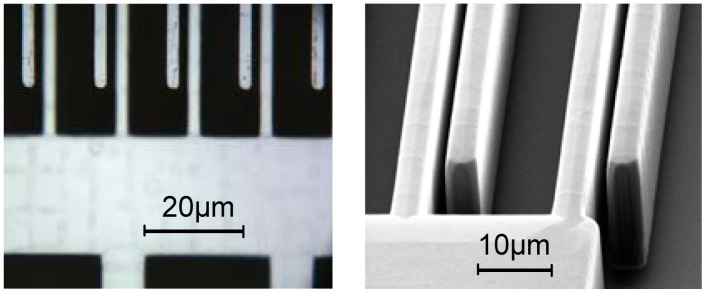
The gap measurements using microscopy. (**a**) Microscopy image; (**b**) SEM image.

**Figure 11.**
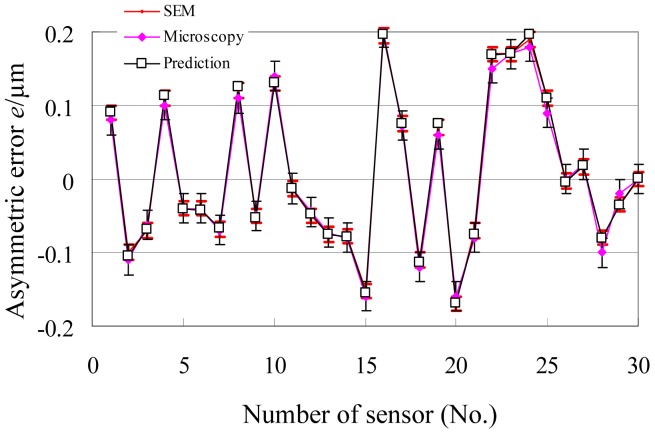
The measurement results of thirty sensors.

Therefore, the predicted relative error that arises from the tested accelerometer is about 4 × 10^−2^, and the absolute error is about 0.16 μm at an average gap of 4 μm. Under common conditions of engineering design, 30 sensors are tested and predicted. The results are shown in Figure 9. The range of the predicted errors is about ± 5 × 10^−2^, and that of the absolute error is about ±0.2 μm under a 4 μm gap. For verification, those sensors are investigated by optical microscopy and SEM, from which the finger images are shown in Figure 10. The measurement results of gap asymmetry are all in the range of ±0.2 μm, even considering the measuring error (Figure 11).

## 4. Conclusions

A fabrication error prediction technique based on a mature MEMS device is presented in this paper. The combined analytical and reverse calibration methods enable direct and clear gap error prediction. Reverse calibration is suitable for deriving the relationship, and the analytical relationship provides the guidelines for rational calibration. In addition, the absence of a quadric term improves prediction precision.

The ratio of the prediction coefficients eliminates the need to calculate the values of parameters, which are difficult to determine. The prediction indicates that the range of relative asymmetric error is about ±5 × 10^−2^, and that the absolute error is about ±0.2 μm under a 4 μm gap. These results coincide with the values measured by optical microscopy and SEM.

## Figures and Tables

**Table 1.** Parameters of the accelerometer system.

**Parameters**	**Values**
DC voltage (*V_d_*) (V)	4.5
Coefficient (*M*_0_)	0.4
Ratio of Stiffness (R)	0.1906
Initial gap designed (μm)	4
